# Another Dental Journal

**Published:** 1886-11

**Authors:** 


### ANOTHER DENTAL JOURNAL.

With the coming year, R. I. Pearson & Co., of Kansas City, will commence the publication of a first-class monthly periodical, to be called *The Western Dental Journal.* It will be edited by J. D. Patterson, D. D. S., with C. L. Hungerford, D. D. S., and A. H. Thompson, D. D. S., as associate editors.

It will be remembered that, during the last year of the existence of the *Missouri Dental Journal,* it was edited by Dr. Pearson, who was then in dental practice, and his associates were Drs. Patterson and Hungerford. They gave to the profession an excellent journal, and it was a matter of regret when they retired. Since then Dr. Pearson has embarked in dental mercantile business, but his love for dental journalism yet remains. When he determined to found another journal, he naturally sought out his old associates for the editorial work, for they had proved their ability and enthusiasm. Should Dr. Thompson join the editorial staff, he will add materially to the *prestige* of the new journal, for he is known as one of the best writers in the profession.

"Westward the star of empire takes its way," and Kansas City is no longer upon the frontier, but has become a great inland city, the center of a wide stretch of wonderful country, and the possibilities of its development no man can foresee. It is no longer inhabited by a back-woods community, and its dentists are fully abreast the advance of thought in professional matters. They need a dental journal of their own, and the prospect is that they will have a good one. We shall heartily welcome the new-comer, and hope it will be well supported by those to whose interests it will be devoted.

